# Clinical data and basal gonadotropins in the diagnosis of central precocious puberty in girls

**DOI:** 10.1530/EC-20-0651

**Published:** 2021-01-06

**Authors:** Teodoro Durá-Travé, Fidel Gallinas-Victoriano, María Malumbres-Chacon, Lotfi Ahmed-Mohamed, María Jesús Chueca -Guindulain, Sara Berrade-Zubiri

**Affiliations:** 1 Department of Pediatrics, School of Medicine, University of Navarra, Pamplona, Spain; 2 Department of Pediatrics, Navarra Hospital Complex, Pamplona, Spain; 3 Navarra Institute for Health Research (IdisNA), Pamplona, Spain

**Keywords:** bone age, central precocious puberty, premature thelarche, predictors of puberty, unstimulated luteinizing hormone, gonadotropin-releasing hormone stimulation test

## Abstract

**Objective:**

The objective of this study was to analyze whether some auxological characteristics or a single basal gonadotropin measurement will be sufficient to distinguish the prepubertal from pubertal status.

**Methods:**

Auxologycal characteristics were recorded and serum LH and FSH were measured by immunochemiluminescence assays before and after GnRH stimulation test in a sample of 241 Caucasian girls with breast budding between 6- and 8-years old. Peak LH levels higher than 5 IU/L were considered a pubertal response. Area under the curve, cut-off points, sensitivity, and specificity for auxologycal variables and basal gonadotropins levels were determined by receiver operating curves.

**Results:**

There were no significant differences in age at onset, weight, height, BMI and height velocity between both groups. Bone age was significantly higher in pubertal girls (*P* < 0.05), although with limited discriminatory capacity. The sensitivity and specificity for the basal LH levels were 89 and 82%, respectively, for a cut off point of 0.1 IU/L. All girls in the pubertal group had a basal LH higher than 1.0 IU/L (positive predictive value of 100%). There was a wide overlap of basal FSH and LH/FSH ratio between prepubertal and pubertal girls.

**Conclusions:**

Auxologycal characteristics should not be used only in the differential diagnosis between prepubertal from pubertal status in 6- to 8-year-old girls. We found a high specificity of a single basal LH sample and it would be useful for establishing the diagnosis of puberty in this age group, reducing the need for GnRH stimulation testing.

## Introduction

Central precocious puberty (CPP) is defined as the premature activation of the hypothalamic–pituitary–gonadal axis (HPG) with the onset of breast development before 8 years of age in girls and an increase in testicular size in boys younger than 9 years of age that is progressive and accompanied by advancement of skeletal age and accelerated linear growth (1, 2, 3). The phenomenon is much more common in girls than in boys (4, 5). On the other hand, idiopatic premature thelarche (PT) is relatively frequent and refers to the isolated development of the breasts before the age of 8 in girls without the activation of the HPG axis. Although most girls with premature thelarche will show spontaneous regression, there is a possibility of progression to true precocious puberty; therefore, it would be mandatory to discriminate between the puberty (precocious puberty) and prepubertal (premature thelarche) values of gonadotropins (6).

Laboratory measurement of gonadotropins, in particular luteinizing hormone (LH), after stimulation with GnRH or GnRH analog (GnRHa) is the standard method to confirm HPG activity (2, 3, 7). Currently, the value of 5 IU/L is accepted as the cut-off point when LH is determined by immunochemiluminescence assay (8, 9, 10, 11, 12). However, the development of additional sensitive immunoassays that measure gonadotropins in serum has motivated several authors to consider the utility of basal LH as a screening method (9, 13, 14, 15).

The objective of this study was to analyze whether some auxological characteristics or a single basal gonadotropin measurement will be sufficient to distinguish the prepubertal from pubertal hypothalamic–pituitary–gonadal axis in girls with breast budding between 6 and 8-years old; and therefore the current method of gonadotropin response to GnRH stimulation could be omitted in many cases.

## Materials and methods

### Participants

This is a retrospective study carried out in a sample of 241 Caucasian girls aged 6 to 8-years who were evaluated for early signs of puberty, such as breast budding, in the Pediatric Outpatient Endocrine Clinic of the Navarra Hospital Complex in Pamplona, Spain, between years 2015 and 2019. Breast development was assessed by both inspection and palpation and rating according to Tanner stage. All participants presented with Tanner stage 2 in breast development (appearance of the breast bud), and were followed up to at least 9 years of age.

The sample of girls was divided into two groups according to the GnRHa stimulation test results. Girls who had a peak LH values ≥5 IU/L were considered as having a pubertal activation of the HPG axis (CPP group), and girls who had lower values were considered prepubertal or premature thelarche (PT group). Patients previously diagnosed with gonadotropin-independent or peripheral precocious puberty were excluded.

Information recorded from every patient included family (paternal and maternal height, age of maternal menarche) and personal data (age at onset of breast budding, weight and height, BMI, and bone age), and GnRHa-stimulation testing results. Weight and height of all the participants had been previously recorded by the pediatrician at the Primary Health Care Center (periodical health checkup), and allowed the calculation of growth velocity 6–12 months before the current clinical evaluation. Target height was calculated in centimeters using the formula: (maternal height + paternal height − 13)/2. Bone age (BA) and height-prognosis was determined using RUS-TW2 method (16).

Weight and height measurements were taken with participants wearing only undergarments and barefoot. Weight was measured using an Año-Sayol scale (reading interval 0–120 kg and a precision of 100 g), and height was measured using a Holtain wall stadiometer (reading interval 60–210 cm, precision 0.1 cm). BMI was calculated according to the following formula: weight (kg)/height^2^ (m).

The SDS values for the weight, height, BMI, and HV were estimated by applying the program Aplicación Nutricional, from the Spanish Society of pediatric gastroenterology, hepatology and nutrition (Sociedad Española de Gastroenterología, Hepatología y Nutrición Pediátrica, available at https://www.gastroinf.es/nutritional/). The graphics from Ferrández *et al.* (Centro Andrea Prader, Zaragoza 2002) were employed as reference charts (17).

### GnRH test

GnRH test was accomplished at least once after 4 months of follow-up in both groups. GnRH stimulation test was performed by determining serum LH and FSH at baseline (between 8:00 h and 9:00 h after an overnight fast) and 4 h post subcutaneous administration of GnRH analog (leuprorelin, 500 µg). LH and follicular stimulating hormone (FSH) were measured by highly sensitive immunochemiluminescence assays (Immulite 2500) with a sensitivity of <0.1 U/L for LH and FSH. Intra-assay coefficients of variation at 0.3 IU/L for LH were 3.5% and FSH 5%. Baseline LH/FSH ratio and LH/FSH ratio post leuprorelin administration were calculated.

### Statistical analysis

The parametric Student’s *t*-test was used to compare the differences in variables recorded between PT and CPP groups. The receiver operating curves (ROC) were constructed after using the exact logistic regression models in order to evaluate the sensitivity and specificity of the auxological variables and basal gonadotropin levels based on predicted probability, and the area under the curve (AUC) was measured for each curve. Youden’s J index, defined as ((sensitivity specificity) - 1), was used to determine the optimal cut point from the ROC curves to discriminate between PT from CPP girls. Statistical analyses were performed using the program Statistical Packages for the Social Sciences version 20.0 (Chicago, IL, USA). Statistical significance was accepted when *P*-value was <0.05.

Parents and/or legal guardians were appropriately informed and gave consent for the participation of the participants in this study in all cases. This study was approved by the Ethics Committee for Human Investigation of the Navarra Hospital Complex, Pamplona, Spain (code: 19/07) in accordance with the ethical standards laid down in the 1964 Declaration of Hensinki and later amendments.

## Results

Table 1 shows and compares mean values of family and personal data registered between PT (prepubertal) and CPP girls. Bone age advancement (BA-CA) are significantly higher in CPP girls (*P* < 0.05). There were no significant differences in target height, height prognosis, and maternal menarche, age at onset, weight, height, and BMI and height velocity between both groups.

**Table 1:** Family and personal data of TP (prepubertal) and CPP girls (M ± SDS).

Items	PT girls (*n* = 133)	CPP girls (*n* = 108)	*P* values*
Target height (cm)	162.0 ± 5.1	161.1 ± 5.5	0.227
Height prognosis (cm)	167.5 ± 4.9	166.5 ± 5.2	0.238
Maternal menarche (years)	12.1 ± 1.5	11.8 ± 1.3	0.464
Age at onset (years)	7.3 ± 0.4	7.4 ± 0.3	0.238
Weight (kg)	30.4 ± 6.6	31.0 ± 6.0	0.52
Weight Z-score	1.02 ± 0.7	1.03 ± 0.9	0.47
Height (cm)	130.3 ± 7.6	132.1 ± 8.2	0.218
Height Z-score	1.23 ± 1.04	1.36 ± 1.2	0.367
BMI (kg/m^2^)	18.1 ± 2.7	17.4 ± 2.4	0.09
BMI Z-score	0.83 ± 0.9	0.91 ± 0.9	0.48
Height velocity (cm/year)	6.8 ± 1.3	7.0 ± 1.6	0.666
Height velocity Z-score	1.67 ± 1.41	1.90 ± 1.73	0.272
BA-CA (years)	1.61 ± 0.67	1.82 ± 0.71	0.024

*Student’s *t*-test.

BA, bone age; CA, chronological age; CPP, central precocious puberty; TP, thelarche premature.

The AUCs for bone age advancement (0.59, 95% CI: 0.51–0.67, *P* = 0.016) were significantly higher than 0.5. Maximal Youden’s J index was reached based on specificity of 52% and sensitivity of 64% for bone age advancement, and bone age advancement cut off point of 1.65 years was chosen to discriminate between PT (prepubertal) and CPP girls. In fact, 69.5% of the girls in the CPP group had a bone age advancement higher than 1.65 years (positive predictive value), while 52.6% of the girls in the prepubertal group had a bone age advancement lower than 1.65 years (negative predictive value).

Table 2 displays and compares the average values for basal and stimulated serum concentrations of gonadotropins (LH, FSH, and LH/FSH ratio) between PT (prepubertal) and CPP girls. Both basal LH and FSH and LH/FSH ratio means as well as the stimulated concentration of LH and FSH and the LH/FSH ratio were significantly higher in the CPP group than in the PT group (*P* < 0.01).

**Table 2:** Basal and stimulated serum concentrations of gonadotropin of PT (prepubertal) and CPP girls (M ± SDS).

Hormone	PT girls (*n* = 133)	CPP girls (*n* =108)	*P* values*
Basal LH (UI/L) (range)	0.06 ± 0.09 **(0.01–1.03)**	0.61 ± 0.94 **(0.01–4,14)**	0.001
LH peak (UI/L) (range)	2.20 ± 1.14 (0.01–4.75)	**12.89 ± 5.34** **(6.09–28.42)**	0.001
Basal FSH (UI/L) (range)	1.49 ± 0.92 **(0.10–4.85)**	3.34 ± 1.82 **(0.44–0.91)**	0.001
FSH peak (UI/L) (range)	15.03 ± 6.48 **(0.27–29.53)**	18.90 ± 6.73 **(8.24–34.71)**	0.001
Basal LH/FSH ratio (range)	0.042 ± 0.388 **(0.01–0.24)**	0.16 ± 0.155 **(0.01–0.8)**	0.001
LH peak/FSH peak ratio (range)	0.156 ± 0.131 **(0.04–1.36)**	0.755 ± 0.53 **(0.19–3.30)**	0.001

Values in bold (except 12.89 ± 5.34) correspond to the range (minimum value and maximum value) *Student’s *t*-test.

**Figure 1:**
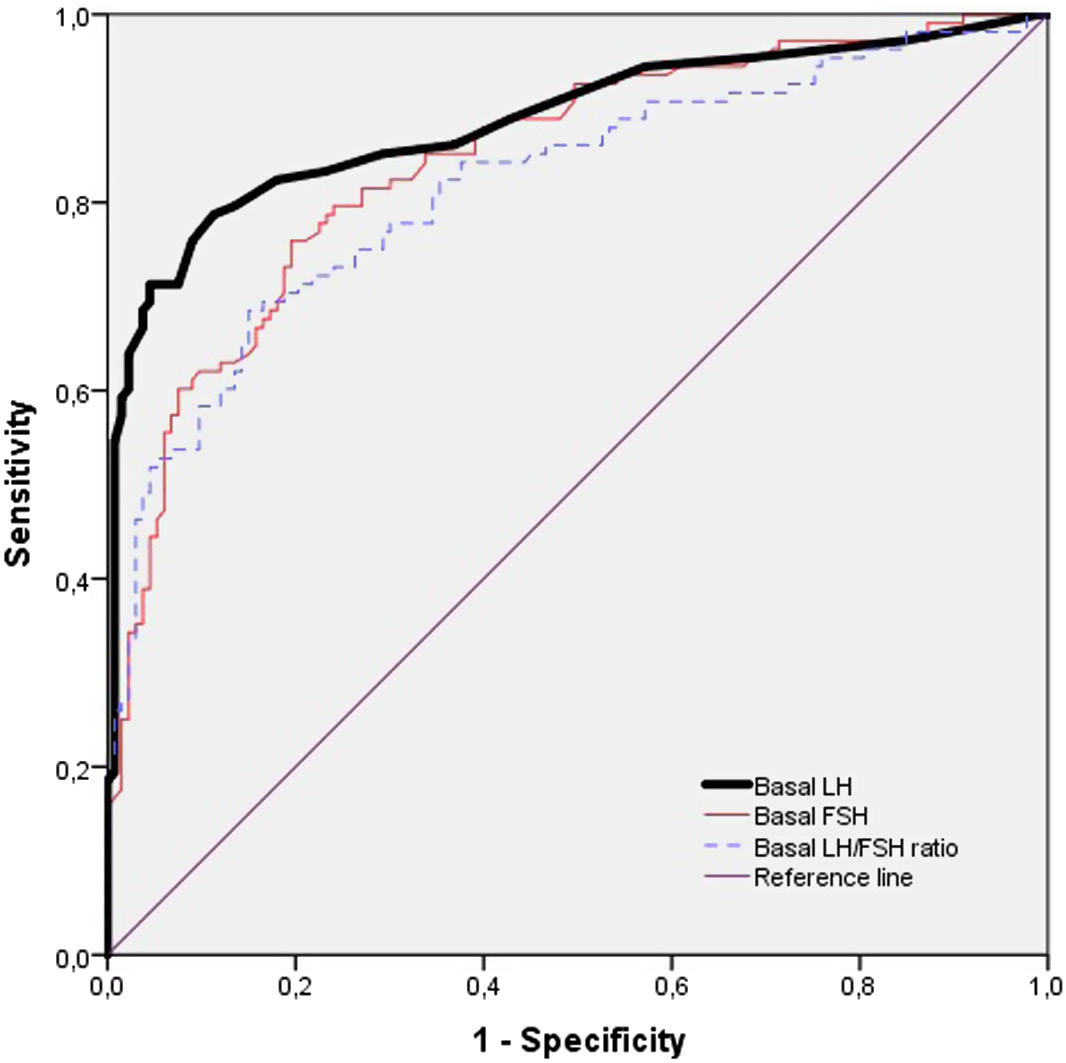
ROC curves used to evaluate the sensitivity and specificity of basal gonadotropin (LH, FSH and LH/FSH ratio).

The AUCs and the optimal cut off point for discriminating PT (prepubertal) from CPP girls using a non-stimulated gonadotropin sample, (basal LH, FSH, and LH/FSH ratio) and based on sensitivity and specificity are shown in Table 3. The AUCs for basal LH, FSH and LH/FSH ratio were significantly higher than 0.5, but the maximum predictability was reached using the basal LH with an AUC of 0.89 (Fig. 1). Maximal Youden’s J index was reached based on specificity of 82% and sensitivity of 89% for basal LH, and LH cut off point of 0.1 IU/l was chosen to discriminate between CPP and prepubertal girls. In fact, 83% of the girls in the CPP group had a basal LH higher than 0.1 IU/L (positive predictive value), while 85% of the girls in the prepubertal group had a basal LH lower than 0.1 IU/L (negative predictive value). All girls in the CPP group had a basal LH higher than 1.0 IU/L (positive predictive value of 100%). The basal FSH yielded less favorable performances such as sensitivity of 63% and specificity of 80% for a cut off point of 2.5 IU/L. The sensitivity and specificity for the basal LH/FSH ratio values were 73 and 74%, respectively, for a cut off point of 0.05. That is, there was a wide overlap of basal FSH and LH/FSH ratio between prepubertal and pubertal girls.

**Table 3:** Sensitivity and specificity for selected cut off point values of basal gonadotropin level discriminating CPP from PT girls, calculated by ROC.

Hormone	AUC (95% CI)	*P*	Cut off point values	Sensitivity (95% CI)	Specificity (95% CI)
LH	0.89 (0.84–0.93)	0.001	0.1	0.82 (0.76–0.88)	0.90 (0.83–0.97)
FSH	0.80 (0.75–0.85)	0.001	2.5	0.63 (0.53–0.73)	0.80 (0.73–0.87)
LH/FSH ratio	0.82 (0.76–0.88)	0.001	0.05	0.73 (0.63–0.83)	0.74 (0.66–0.82)

## Discussion

This study features that auxological characteristics represent a weak predictor for GnRH-dependent PP and should not be used only in the differential diagnosis between PT and CPP in 6- to 8-year-old girls. However, we found a high specificity of a single basal LH sample and it would be useful for establishing the diagnosis of CPP in this age group.

Most cases of premature thelarche present in the first four years of life and regress before puberty, but girls who present later may be accompanied by accelerated growth and advanced bone age. The name given to this condition by several authors was ‘slowly progressive variant of precocious puberty’ associated with both basal and GnRH-stimulated serum LH levels of prepubertal characteristics (2, 10). Our data show that most of the auxological characteristics recorded overlapped between girls with PT and CPP aged 6–8 years. In fact, there were no significant differences in weight, height, BMI, and growth velocity between both groups; only advanced bone age emerged as a significant predictor of CPP in this age group, although with limited discriminatory capacity (positive predictive value: 69.5%). Therefore, an exclusive measurement of the auxological characteristics would not be sufficient to diagnose or exclude CPP in girls with breast budding in this age range (18). In addition, it should be noted that the prognosis of adult height of the CPP girls, which at the time of diagnosis is usually overvalued by clinical and auxological conditions (19), did not differ from that calculated for the PT girls.

Diagnosis of CPP is based on clinical evaluation and laboratory hormonal assessment. Different hormone assays with high sensitivity to measure gonadotropins levels are currently available, such as immunofluorometric, immunochemiluminiscence (ICMA) or electrochumiluminescence. But ICMA is the most commonly used – it has also been used in this study – and allows reasonable discrimination between prepubertal and pubertal status using a single basal LH level (11). The distinct cut-off values of basal LH assessed by ICMA that indicate HPG axis activation varies according to the different authors, and oscillate between 0.1 and 1.1 IU/L (9, 10, 13, 20, 21). Caution should be used when interpreting gonadotropin concentrations in girls in the first three years of life, because basal gonadotropin concentrations are usually high in this age group (22). Our results, carried out in a larger cohort in girls aged 6–8 years, confirm the high sensitivity (82%) and specificity (90%) of a single basal LH sample for establishing the diagnosis of CPP when basal LH sample was higher than 0.1 IU/L, with a positive predictive value of 83%. However, the positive predictive value of 100% obtained in this study from a single baseline LH sample was obtained with a cut-off point of 1.0 IU/L. Since the sensitivity and specificity of a single basal LH sample for the diagnosis of CPP varies in relation to cut-off value and laboratory methodology (11, 12), several authors have recommended blood sample collection to obtain normal basal values of gonadotropins in clinical centers that care for girls with CPP (7, 8, 9).

As both gonadotropin determinations (basal LH, FSH and LH/FSH ratio) are supposed to be increased in the HPG axis activation, they have been routinely measured during the evaluation of CPP and, in fact, both have discriminatory capacity between CPP and prepubertal girls. However, as several authors have described (9, 10, 13, 14, 15), this study found that the measurement of basal FSH concentrations or basal LH/FSH ratio do not improve diagnostic sensitivity over basal LH alone in girls aged 6–8 years undergoing evaluation for CPP. This lower discriminatory capacity observed in basal FSH and LH/FSH ratio seems to derive from the overlap of the FSH levels observed in girls with and without CPP. Serum levels of estradiol are not used to diagnose CPP, considering their low sensitivity and large overlap between normal prepubertal and pubertal children (11). Nevertheless, novel laboratory techniques such as tandem mass spectrometry might improve the sensitivity and specificity of estradiol assays (23). In fact, different authors have described that GnRHa-stimulated serum estradiol level at 24 h may be a useful indicator of pubertal activation, regardless of the LH values (21, 24). However, GnRH-stimulated serum LH level provides the best clinical model due to its practicality and convenience when evaluating puberty in girls.

**Figure 2:**
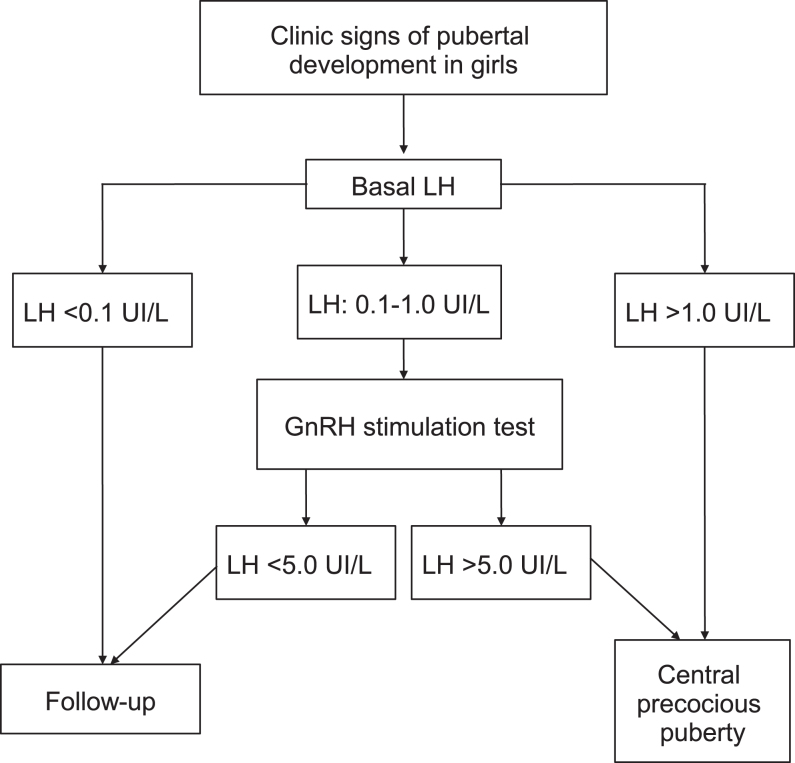
Algorithm proposed for the diagnosis of central precocious puberty.

In the present study, we found that a basal LH level greater than 0.1 IU/L would be suggestive of central pubertal activation (positive predictive value of 83%) in those girls who have breast budding accompanied by physical suspicion of precocious puberty (accelerated growth rate and, especially, bone age advancement). However, if LH level was greater than 1.0 IU/L (positive predictive value of 100%), a diagnosis of CPP can be made, avoiding the inconvenience, costs and time of the GnRH stimulation test. Thus, a sample for basal LH measurement could be obtained by the primary care physician for initial management: a LH level higher than 0.1 IU/L, would mean a reasonable suspicion of puberty status that should be confirmed by GnRH stimulation; if basal LH level was higher than 1.0 IU/L, the girl should be referred for additional assessment concerning the etiology of precocious puberty and to determine adequate treatment by the pediatric endocrinologist. In contrast, baseline LH levels are not sufficiently sensitive to rule out CPP in girls whose baseline LH levels are <0.1 IU/L; furthermore, even if the basal levels of LH were undetectable in the presence of clinical signs of progressive pubertal development, the stimulation test would be necessary for the identification of hypothalamic–pituitary–gonadal axis activation. In this way, an algorithm for the diagnosis of CPP was proposed for our endocrinology unit (Fig. 2) based on the data obtained in this study. Patients should be followed on a quarterly basis until at least 9 years of age in order to exclude possible advanced puberty.

There are certain limitations in the diagnostic sensitivity of basal LH levels. Indeed, 17% of the girls who had CPP in our cohort had an initial LH concentration of <0.1 IU/L. This absence of concordance between the GnRHa stimulation tests with the basal level of LH could be due to the fact that the girls had been evaluated at an early stage of puberty, since in the first months of pubertal development hypothalamic–pituitary–gonadal axis activation is progressive and even oscillating (10). In fact, in 15 cases out of the 108 girls with CPP included in this study (14%), a previous GnRHa test was performed with a prepubertal result, but with a subsequent pubertal progression. However, it is possible that some of our patients who presented with PT represent slowly progressing variants of CPP (2). Another limitation is that girls with peripheral sexual precocity have suppressed basal LH levels. That is, the presence of a high concentration of estradiol with low basal gonadotropins together with a rapid development of secondary sexual characteristics should compel to investigate for peripheral causes (adrenal or ovarian tumors, exposure to exogenous sex steroids, etc.). Furthermore, due to the diurnal fluctuation of gonadotropin levels, especially in early puberty, the evaluation of basal serum LH levels would be debatable (15). Despite these restrictions, we found that a basal LH level greater than 0.1 UI/L in girls is highly suggestive of central pubertal activation, whereas an undetectable LH value does not exclude a GnRHa test.

In conclusion, we support that, in the majority of girls aged 6 to 8 years presenting with premature thelarche, a single basal LH determination would be of high diagnostic usefulness in identifying girls who have CPP, so reducing the need for GnRH stimulation testing. Basal FSH concentrations or basal LH/FSH ratio do not improve diagnostic sensitivity in girls undergoing evaluation for CPP. However, clinical judgment and follow-up continue to be of greater importance in the evaluation of this entity.

## Declaration of interest

The authors declare that there is no conflict of interest that could be perceived as prejudicing the impartiality of the research reported.

## Funding

This work did not receive any specific grant from any funding agency in the public, commercial, or not-for-profit sector.

## Author contribution statement

T D T and F G V participated in study design and data analysis, and wrote the first draft of the manuscript. M M C, L A M, S B Z and M C G participated in data collection and analysis. All authors participated in manuscript preparation and approved its final version.

## References

[1] Parent AS Teilmann G Juul A Skakkebaek NE Toppari J Bour-guignon JP The timing of normal puberty and the age limits of sexual precocity: variations around the world, secular trends, and changes after migration. Endocrine Reviews 2003 24 668–693. (10.1210/er.2002-0019) 14570750

[2] Dattani MT Hindmarsh PC Normal and abnormal puberty. In Clinical Pediatric Endocrinology, ch. 10, 5th ed., pp. 183–210. Eds Brook CGD Clayton PR Brown RSBoston, MA, USA: Blackwell Publishing, 2005.

[3] Carel JC Léger J Clinical practice. Precocious puberty. New England Journal of Medicine 2008 358 2366–2377. (10.1056/NEJMcp0800459) 18509122

[4] Soriano-Guillén L Corripio R Labarta JI Cañete R Castro-Feijóo L Espino R Argente J Central precocious puberty in children living in Spain: incidence, prevalence, and influence of adoption and immigration. Journal of Clinical Endocrinology and Metabolism 2010 95 4305–4313. (10.1210/jc.2010-1025) 20554707

[5] Rohani F Salehpur S Saffari F Etiology of precocious puberty, 10 years study in Endocrine Reserch Centre (Firouzgar), Tehran. Iranian Journal of Reproductive Medicine 2012 10 1–6. 25242967 PMC4163256

[6] Varimo T Huttunen H Miettinen PJ Kariola L Hietamäki J Tarkkanen A Hero M Raivio T Precocious puberty or premature thelarche: analysis of a large patient series in a single tertiary center with special emphasis on 6- to 8-year-old girls. Frontiers in Endocrinology 2017 8 213. (10.3389/fendo.2017.00213) PMC5572337 28878739

[7] Bangalore Krishna K Fuqua JS Rogol AD Klein KO Popovic J Houk CP Charmandari E Lee PA Freire AV Ropelato MG ***et al***. Use of gonadotropin-releasing hormone analogs in children: update by an international consortium. Hormone Research in Paediatrics 2019 91 357–372. (10.1159/000501336) 31319416

[8] Mogensen SS Aksglaede L Mouritsen A Sørensen K Main KM Gideon P Juul A Diagnostic work-up of 449 consecutive girls who were referred to be evaluated for precocious puberty. Journal of Clinical Endocrinology and Metabolism 2011 96 1393–1401. (10.1210/jc.2010-2745) 21346077

[9] Pasternak Y Friger M Loewenthal N Haim A Hershkovitz E The utility of basal serum LH in prediction of central precocious puberty in girls. European Journal of Endocrinology 2012 166 295–299. (10.1530/EJE-11-0720) 22084156

[10] Harrington J Palmert MR Hamilton J Use of local data to enhance uptake of published recommendations: an example from the diagnostic evaluation of precocious puberty. Archives of Disease in Childhood 2014 99 15–20. (10.1136/archdischild-2013-304414) 24170688

[11] Brito VN Spinola-Castro AM Kochi C Cristiane Kopacek C Alves da Silva PC Guerra-Junior G Central precocious puberty: revisiting the diagnosis and therapeutic management. Archives of Endocrinology and Metabolism 2016 60 163–172. (https://doi:) 27191050 10.1590/2359-3997000000144

[12] Latronico AC Brito VN Carel JC Causes, diagnosis, and treatment of central precocious puberty. Lancet: Diabetes and Endocrinology 2016 4 265–274. (10.1016/S2213-8587(1500380-0) 26852255

[13] Houk CP Kunselman AR Lee PA Adequacy of a single unstimulated luteinizing hormone level to diagnose central precocious puberty in girls. Pediatrics 2009 123 e1059–e1063. (10.1542/peds.2008-1180) 19482738

[14] Lee HS Park HK Ko JH Kim YJ Hwang JS Utility of Basal luteinizing hormone levels for detecting central precocious pubertyin girls. Hormone and Metabolic Research 2012 44 851–854. (10.1055/s-0032-1321905) 22893259

[15] Carretto F Salinas-Vert I Granada-Yvern ML Murillo-Vallés M Gómez-Gómez C Puig-Domingo M Bel J The usefulness of the leuprolide stimulation test as a diagnostic method of idiopathic central precocious puberty in girls. Hormone and Metabolic Research 2014 46 959–963. (10.1055/s-0034-1387790) 25295414

[16] Tanner JM Whitehouse RH Marshall WA Healy MJR Goldstein H Assessment of Skeletal Maturity and Prediction of Adult Height (TW2 Method), 2nd ed. London: Academic Press, 1983.

[17] Ferrández A Baguer L Labarta JI Labena C Mayayo E Puba B Rueda C Ruiz-Echarri M Longitudinal pubertal growth according to age at pubertal study of normal Spanish children from birth to adulthood. Pediatric Endocrinology Reviews 2005 2 423–559.

[18] Lee DM Chung IH Morning basal luteinizing hormone, a good screening tool for diagnosing central precocious puberty. Annals of Pediatric Endocrinology and Metabolism 2019 24 27–33. (10.6065/apem.2019.24.1.27) 30943677 PMC6449618

[19] Durá-Travé T Ortega-Pérez M Ahmed-Mohamed L Moreno-González P Chueca-Guindulain MJ Berrade-Zubiri S Central precocious puberty in girls: diagnostic study and auxological response to triptorelin treatment. Endocrinología, Diabetes y Nutrición 2019 6 410–416. (https://doi:) 10.1016/j.endinu.2018.12.007 30808564

[20] Neely EK Hintz RL Wilson DM Lee PA Gautier T Argente J Stene M Normal ranges for immunochemiluminometric gonadotropin assays. Journal of Pediatrics 1995 127 40–46. (10.1016/s0022-3476(9570254-7) 7608809

[21] Sathasivam A Garibaldi L Shapiro S Godbold J Rapaport R Leuprolide stimulation testing for the evaluation of early female sexual maturation. Clinical Endocrinology 2010 73 375–381. (10.1111/j.1365-2265.2010.03796.x) 20184599

[22] Bizzarri C Spadoni GL Bottaro G Montanari G Giannone G Cappa M Cianfarani S The response to gonadotropin releasing hormone (GnRH) stimulation test does not predict the progression to true precocious puberty in girls with onset of premature thelarche in the first three years of life. Journal of Clinical Endocrinology and Metabolism 2014 99 433–439. (10.1210/jc.2013-3292) 24297793

[23] Ketha H Kaur S Grebe SK Singh RJ Clinical applications of LC-MS sex steroid assays: evolution of methodologies in the 21st century. Current Opinion in Endocrinology, Diabetes, and Obesity 2014 21 217–226. (10.1097/MED.0000000000000068) 24739314

[24] Chin VL Cai Z Lam L Shah B Zhou P Evaluation of puberty by verifying spontaneous and stimulated gonadotropin values in girls. Journal of Pediatric Endocrinology and Metabolism 2015 28 387–392. (10.1515/jpem-2014-0135) 25514323 PMC4767152

